# Spontaneous Phase Segregation Enabling Clogging Aversion in Continuous Flow Microfluidic Synthesis of Nanocrystals Supported on Reduced Graphene Oxide

**DOI:** 10.3390/nano12234315

**Published:** 2022-12-05

**Authors:** Dumei Wang, Dongtang Zhang, Yanan Wang, Guangsheng Guo, Xiayan Wang, Yugang Sun

**Affiliations:** 1 Center of Excellence for Environmental Safety and Biological Effects, Beijing Key Laboratory for Green Catalysis and Separation, Department of Chemistry and Biology, Beijing University of Technology, Beijing 100124, China; 2 Minzu University of China, Beijing 100081, China; 3 Department of Chemistry, Temple University, 1901 North 13th Street, Philadelphia, PA 19122, USA

**Keywords:** solid-phase segregation in liquid, hydrophilicity to hydrophobicity, liquid-solid segmented flow, continuous flow microfluidic synthesis, nanoparticles supported on reduced carbon

## Abstract

Eliminating clogging in capillary tube reactors is critical but challenging for enabling continuous-flow microfluidic synthesis of nanoparticles. Creating immiscible segments in a microfluidic flow is a promising approach to maintaining a continuous flow in the microfluidic channel because the segments with low surface energy do not adsorb onto the internal wall of the microchannel. Herein we report the spontaneous self-agglomeration of reduced graphene oxide (rGO) nanosheets in polyol flow, which arises because the reduction of graphene oxide (GO) nanosheets by hot polyol changes the nanosheets from hydrophilic to hydrophobic. The agglomerated rGO nanosheets form immiscible solid segments in the polyol flow, realizing the liquid–solid segmented flow to enable clogging aversion in continuous-flow microfluidic synthesis. Simultaneous reduction of precursor species in hot polyol deposits nanocrystals uniformly dispersed on the rGO nanosheets even without surfactant. Cuprous oxide (Cu_2_O) nanocubes of varying edge lengths and ultrafine metal nanoparticles of platinum (Pt) and palladium (Pd) dispersed on rGO nanosheets have been continuously synthesized using the liquid–solid segmented flow microfluidic method, shedding light on the promise of microfluidic reactors in synthesizing functional nanomaterials.

## 1. Introduction

Nanosheets of graphene and reduced graphene oxide (rGO) have been widely used as a class of promising support for catalyst nanoparticles due to their unique mechanical flexibility/strength and electronic structures [1,2,3]. Loading nanoparticles on rGO nanosheets can be accomplished through the self-assembly of nanoparticles on the rGO nanosheets driven by specific interactions, including electrostatic attraction and hydrophobic-hydrophobic interactions [4,5]. Impregnation of rGO nanosheets with nanoparticle precursor chemicals followed by appropriate thermal treatment and/or chemical reactions has also been successfully demonstrated to load nanoparticles on the rGO nanosheets [6]. However, despite the successful progress, batch synthesis approaches face the challenge of continuous operation and mass production. In contrast, microfluidics that possesses merits including continuous and controllable fluid transport, enhanced mass and heat transfer [7], precise manipulation of the reaction parameters [8], and fast screening of experimental conditions with low consumption [9] have been explored as an emerging method to synthesize nanomaterials. For example, researchers have used microfluidic systems to synthesize nanoparticles with controllable size, shape, and structure [10,11]. Unlike batch synthesis, the continuous flow operation and feasibility of integrating multiple parallel microfluidic channels support mass production with continuous flow microfluidic synthesis [12,13].

When nanoparticle synthesis is performed in a microfluidic channel, nuclei and small nanoparticles may easily adsorb onto the inner surface of the microfluidic channel because of the inherently high surface energy originating from ultrahigh surface curvatures of the small nanoparticles. Continuous synthesis would accumulate more small nanoparticles on the microchannel surface, inevitably causing clogging of the microchannel. Eliminating the blockage of microchannels is challenging but critical to maximizing the advantages of continuous-flow microfluidic synthesis. Segmented flow microfluidic reactors have been evaluated to prevent clogging by introducing an additional immiscible fluid or an inert gas into the microchannel to split the reaction phase into discrete plugs or droplets [14,15]. Both gas–liquid and liquid–liquid segmented flow systems have been successfully applied to the production of nanoparticles made of varying compositions, including noble metals [16,17], metal oxides [18], and semiconductors [19]. Introducing the immiscible liquid or inert gas segments to microfluidic flows requires a very precise design of flow channels and mixers and becomes extremely difficult for thin channels and the high flow rate of reaction fluids.

In this study, we develop a spontaneous phase segregation flow strategy that relies on the self-agglomeration of nanoparticle composite to form solid segments in a liquid flow to continuously synthesize rGO-supported nanocrystals at high speed. The performance of this strategy has been evaluated by the successful synthesis of Cu_2_O nanocubes and metal nanoparticles dispersed on rGO nanosheets.

## 2. Materials and Methods

### 2.1. Materials

Graphite flakes, sodium chloride (NaCl, ≥99.0%), sulfuric acid (H_2_SO_4_, 98%), sodium nitrate (NaNO_3_, ≥99%), potassium permanganate (KMnO_4_, ≥99%), hydrogen peroxide (H_2_O_2_, 30% in water), concentrated aqueous solution hydrochloric acid (HCl, 37 wt.%), copper(II) nitrate hydrate (Cu(NO_3_)_2_·3H_2_O, ≥99%), chloroplatinic acid hexahydrate (H_2_PtCl_6_·_6_H_2_O, 99.9%), palladium chloride (PdCl_2_, 99%), ethylene glycol (EG, 99%), and triethylene glycol (TEG, 99%) were purchased from Sinopharm Chemical Reagent Co., Ltd. (Beijing, China). All chemicals were used as received without further purification. Deionized (DI) water was used in this experiment to prepare the solutions.

### 2.2. Synthesis of Cu_2_O Nanocrystals on Reduced Graphene Oxide (rGO) Nanosheets

The graphene oxide (GO) nanosheets were synthesized using Hummers’ method [20], the details are shown in the supplementary material. In a typical synthesis (Schematic S1) of rGO-supported Cu_2_O nanocrystals (Cu_2_O/rGO), 10 mg of GO nanosheets was dispersed in 30 mL of DI water with the assistance of ultrasonication for 30 min. After forming uniform GO dispersion, 0.15 mmol of Cu(NO_3_)_2_·3H_2_O was added to this suspension and stirred for 10 min. Then, the pH value was adjusted to ~5 by adding 200 μL of NaOH aqueous solution (1 M). Stirring the dispersion for 2 h led to the adsorption of Cu^2+^ ions on the GO nanosheets. The GO nanosheets impregnated with Cu^2+^ ions were collected through centrifugation and re-dispersed in 30 mL of TEG containing 2.5 mL of DI water to form the synthesis solution. The re-dispersal process was assisted by ultrasonication (320 W, 40 kHz, 495 × 292 × 152 mm^3^). Finally, the synthesis solution containing GO soaked with Cu^2+^ ions was added to the pressurized container with pressure-regulated nitrogen gas, and the solution flowed into a capillary microfluidic reactor continuously. The microfluidic reactor used a quartz capillary tube (with an inner diameter of 200 μm and an outer diameter of 365 μm). The lengths of the microchannel in the heating region and cooling region were ∼200 cm and 10 cm, respectively. The actual flow rate was ~1 mL/min. The reactions were performed at temperatures ranging from 220 °C to 350 °C. The product solution was collected in a glass vial. The synthesized composite nanoparticles were collected through centrifugation and washing with ethanol three times. The Cu_2_O/rGO composite particles were dried at 60 °C overnight. The Cu_2_O/rGO composite particles synthesized at 220 °C, 250 °C, 280 °C, 300 °C, 330 °C, and 350 °C were denoted as Cu_2_O/rGO-220, Cu_2_O/rGO-250, Cu_2_O/rGO-280, Cu_2_O/rGO-300, Cu_2_O/rGO-330, and Cu_2_O/rGO-350, respectively.

### 2.3. Characterization Methods

X-ray diffraction (XRD) patterns were recorded on a Bruker D8 Advance X-ray diffractometer (Bruker D8, Karlsruhe, Germany) with Cu Kα radiation (*λ* = 1.5406 Å) at 40 kV and 2*θ* ranging from 10° to 80°. Transmission electron microscopy (TEM) images and high-resolution TEM (HRTEM) images were recorded with a JEOL JEM-1400 microscope (JEOL, Tokyo, Japan) and a JEOL 2100 microscope (JEOL, Tokyo, Japan). Selected-area electron diffraction (SAED) patterns were also recorded on the TEM microscopes. Scanning electron microscopy (SEM) images were collected with an FEI Quanta 450 FEG microscope (FEI, Reston, VA, USA) operated at an accelerated voltage of 20 kV and high vacuum mode. Energy-dispersive X-ray spectroscopy (EDS) analysis was conducted on the SEM microscopy equipped with an X-MaxN 50 spectrometer (Oxford Instruments, Abingdon, UK). The samples were prepared by drop-casting an appropriate amount of ethanol dispersion of the products on silicon wafers for XRD measurement, or carbon-coated copper grids for TEM and SEM imaging, followed by drying in a fume hood and at room temperature. Laser scattering of microfluidic flow was recorded using the home-built microscopic system detailed in our previous work [21].

## 3. Results

**Figure 1:**
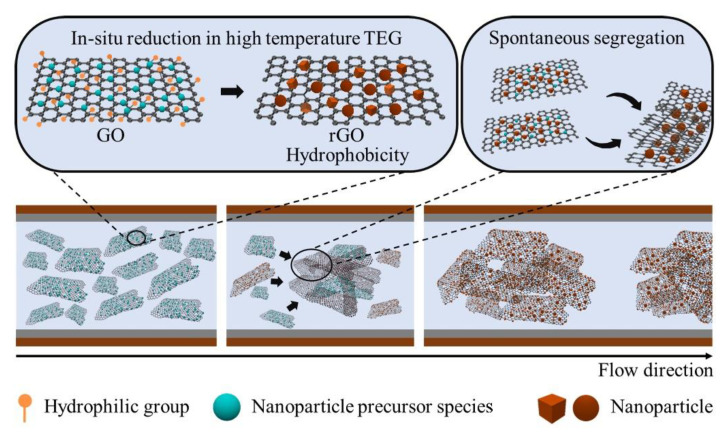
Schematic illustration of the major evolution steps involved spontaneous phase segregation of rGO-supported nanocrystal agglomerates during the simultaneous formation of nanocrystals and reduction of GO in hot polyols.

A schematic representation of the formation of spontaneous phase segregation during the continuous flow microfluidic synthesis is provided in Figure 1. The synthesis solution containing GO nanosheets soaked with nanocrystal precursors is pressurized by N_2_ gas to flow through the microfluidic channel. The high boiling point of TEG solvent ensures TEG is liquid and has continuous flow even under high reaction temperatures. Once the synthesis solution flows to the heating zone, the temperature of the synthesis solution increases to the set temperature rapidly because of the small cross-section of the capillary tube. The elevated temperature increases the reducing ability of TEG to simultaneously reduce the nanocrystal precursor species to form nanocrystals and reduce the GO nanosheets to rGO nanosheets. The transformation of GO to rGO changes the nanosheets from hydrophilic to hydrophobic materials. The hydrophobic rGO nanosheets repel TEG solvent to agglomerate through hydrophobic–hydrophobic interactions and self-segregate into solid plugs in the flowing synthesis solution. The nanocrystals formed from reduction are simultaneously wrapped in the agglomerated rGO nanosheets. The formation of hydrophobic solid plugs from the hydrophilic liquid (TEG) realizes solid–liquid segmented flow, enabling continuous synthesis in capillary microchannels. When the agglomerated rGO-supported nanocrystals are collected and washed, they can be re-dispersed in an appropriate solvent with the assistance of ultrasonication.

**Figure 2:**
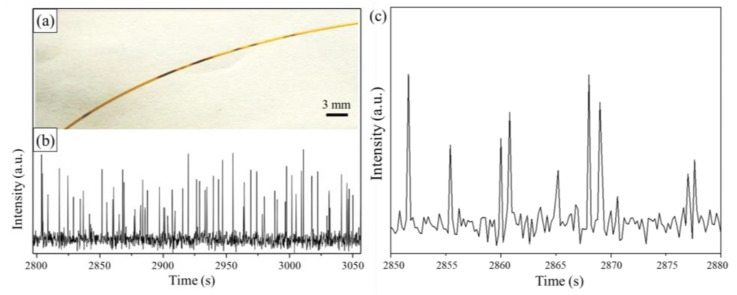
(**a**) Photo of a section of the capillary tube in the cooling zone. (**b**,**c**) Time-dependent laser scattering intensity as the TEG dispersion of GO nanosheets flows in the capillary tube. The temperature in the heating zone was 250 °C.

The formation of solid plugs depends on the temperature of the synthesis solutions, which determines the reduction ability of TEG (Table S1). The solid plugs appear only when the temperature is above 200 °C, indicating that the strength of the reduction power of TEG is critical to reducing the hydrophilic GO nanosheets to form hydrophobic rGO nanosheets. Figure 2a presents a snapshot photo of a microfluidic tube outside the heat zone for the synthesis at 250 °C, showing the presence of a train of black segments in the tube. The observation confirms the agglomeration of rGO nanosheets into solid plugs. When a laser beam is focused on a fixed position of the capillary tube in which the solution containing rGO flows continuously, the place without rGO agglomerate is the baseline, and the passing rGO agglomerate plugs significantly scatter the laser beam to give spikes in the time-dependent signal profile. The variation of the detection signal as a function of time is presented in Figure 2b,c, showing the appearance of sharp spikes originating from the sudden transformation of the flowing phase from liquid solvent to solid plugs. A longer solid plug gives a broader peak, while a sharper peak corresponds to a shorter solid plug. The intensity of a spike peak may reflect the agglomeration density of the solid plug. For example, a denser agglomerate of rGO nanosheets gives a spike peak with higher intensity. The hydrophobicity of the solid rGO plugs prevents them from attaching to the internal wall of the capillary tube. Forming the short rGO segments in TEG liquid is beneficial for continuous flow and prevents clogging of the microfluidic channel.

When the nanocrystal precursor species adsorbed on GO nanosheets are Cu(OH)_2_, the product composite particles are Cu_2_O or Cu nanocrystals dispersed on rGO nanosheets (Cu_2_O/rGO or Cu/rGO) depending on the reaction temperature. The high-density oxygen-containing groups (e.g., carboxyl groups) on the surface of the GO nanosheets provide adsorption sites for Cu^2+^ ions. After the adsorption of Cu^2+^ ions, adjusting the pH of the Cu^2+^/GO dispersion to ~5 transforms the Cu^2+^ ions to copper hydroxide on the GO nanosheets (Cu(OH)_2_/GO). The Cu(OH)_2_/GO nanosheets are collected by filtration to remove excess ionic species and are re-dispersed in TEG to form the synthesis solution. As the synthesis solution flows into the heating zone, the rapid temperature increase in the synthesis solution can quickly reduce the adsorbed Cu(OH)_2_ to trigger heterogeneous nucleation and growth of Cu_2_O nanocrystals on the GO nanosheets, which are also reduced simultaneously, forming Cu_2_O nanocrystals dispersed on rGO nanosheets (Cu_2_O/rGO). In contrast, using water instead of TEG as the solvent, only CuO nanocrystals could be synthesized in such a non-reducing environment (Figure S1). In addition, STEM mapping was used to characterize the element distribution of precursors before entering the channel. As shown in Figure S2, the elements of Cu, C, and O were evenly distributed on the surface of graphene, indicating that the adsorption of Cu^2+^ ions was uniform. Because of the uniform adsorption of Cu^2+^ ions on the GO nanosheets, the Cu_2_O nanocrystals are uniformly dispersed on the rGO nanosheets. The stepwise temperature increase in the microfluidic channel makes it easy to control the supersaturation of Cu(I) intermediate species on the nanosheets and thus control the nucleation of Cu_2_O nanocrystals. At a low reaction temperature, the reduction rate of Cu(OH)_2_ is slow to generate a low supersaturation of Cu(I) intermediate species, resulting in a small number of nuclei. Therefore, the final Cu_2_O nanocrystals formed at a lower temperature are larger for the Cu(OH)_2_/GO with the same loading of Cu^2+^. The reaction at a higher temperature forms more Cu_2_O nuclei and smaller Cu_2_O nanocrystals in the final product. When the temperature is high enough, the reducing ability of TEG becomes strong enough to reduce the Cu(OH)_2_ nanocrystals to metallic Cu.

**Figure 3:**
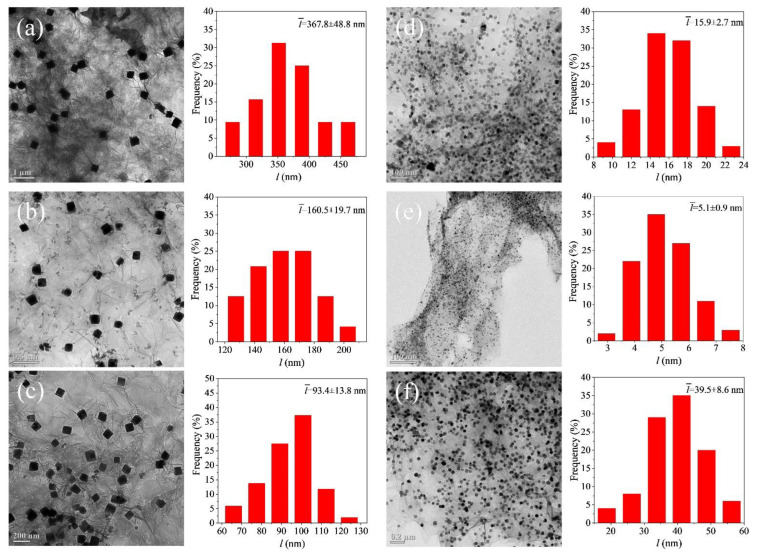
TEM images (left frames) and size histograms (right frames) of the Cu_2_O/rGO composites (i.e., Cu_2_O nanocrystals dispersed on rGO nanosheets) synthesized at different temperatures: (**a**) 220 °C, (**b**) 250 °C, (**c**) 280 °C, (**d**) 300 °C, (**e**) 330 °C, and (**f**) 350 °C. The corresponding products are labeled as (**a**) Cu_2_O/rGO-220, (**b**) Cu_2_O/rGO-250, (**c**) Cu_2_O/rGO-280, (**d**) Cu_2_O/rGO-300, (**e**) Cu_2_O/rGO-330, and (**f**) Cu_2_O/rGO-350, respectively. The statistical histograms of size distributions of the major nanocrystals were determined by analyzing the edge lengths (*l*) of 200 randomly selected nanoparticles. Note: The dominating nanocrystals in the Cu_2_O/rGO-350 sample shown in (**f**) were Cu, even though the sample label was still “Cu_2_O” to show the consistency with the samples synthesized at different temperatures.

**Figure 4:**
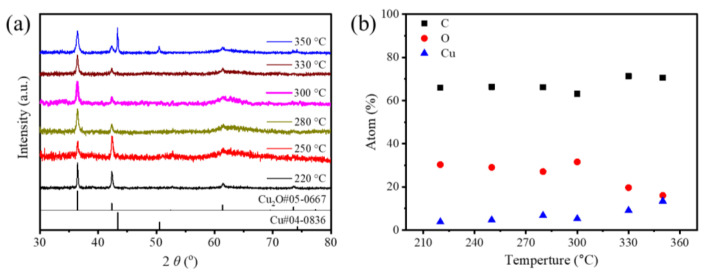
(**a**) XRD patterns and (**b**) elemental compositions of the Cu_2_O/rGO composite particles synthesized at different reaction temperatures.

Figure 3 presents the TEM images of the composite particles synthesized from the solutions containing dispersed Cu(OH)_2_/GO nanosheets of the same amount at different reaction temperatures. The resulting nanocrystals are uniformly dispersed on the rGO nanosheets and exhibit cubic morphologies. XRD patterns of these composite samples indicate that the nanocubes formed at temperatures of 220–330 °C are crystalline Cu_2_O and the nanocubes formed at 350 °C are a mixture of crystalline Cu_2_O and Cu (Figure 4a). The XRD patterns of the Cu_2_O nanocubes exhibit diffraction peaks at 36.4°, 42.3°, and 61.3°, corresponding to the (111), (200), and (220) reflections of cubic Cu_2_O, respectively [22]. The width of the (111) peak of the product samples increases with the reaction temperature, indicating that the edge length of the Cu_2_O nanocubes synthesized at a higher temperature is smaller than that of the Cu_2_O nanocubes synthesized at a lower temperature (see Table S2). Statistical analysis of the Cu_2_O nanocubes shows that their average edge lengths indeed decrease with the reaction temperature, i.e., 367.8 nm, 160.5 nm, 93.4 nm, 15.9 nm, and 5.1 nm for the Cu_2_O synthesized at 220 °C, 250 °C, 280 °C, 300 °C, and 330 °C, respectively (see statistic histograms in Figure 3). Additional sharp XRD peaks appear at 43.3° and 50.4° for the sample synthesized at 350 °C, which are indexed to the (111) and (200) reflections of a face-centered cubic Cu lattice. The formation of Cu nanoparticles confirms that the reducing power of TEG increases with temperature and becomes strong enough at 350 °C to deeply reduce Cu(OH)_2_ to Cu. The nucleation and growth kinetics for transforming Cu(OH)_2_ to Cu nanoparticles are different from that for forming Cu_2_O nanoparticles. The Cu nanoparticles with more rounded cubic morphologies are much larger than the Cu_2_O nanoparticles, which are too small to be resolved in the TEM image. According to the dependence of the size of the Cu_2_O nanocubes on the reaction temperature revealed in Figure 3a–e, the Cu_2_O nanocrystals formed from the synthesis at 350 °C are expected to exhibit sizes smaller than the Cu_2_O nanocubes synthesized at 330 °C (i.e., 5.1 nm). The XRD peak of Cu_2_O (111) reflections for the sample synthesized at 350 °C (full width at half maximum (FWHM) of 0.416 degrees) is broader than that of the sample synthesized from the reaction at 330 °C (FWHM of 0.336 degrees), confirming the smaller size of the Cu_2_O nanocrystals formed at 350 °C. In contrast, the metallic Cu nanoparticles exhibit an average size of 39.5 nm, representing the dominating signals in the TEM image (Figure 3f). The results verify the feasibility of solid–liquid segment flow in synthesizing reduced nanocrystals dispersed on rGO nanosheets when the temperature is high enough to simultaneously reduce the GO nanosheets and precursor species. The composition and dimension of the rGO-supported nanocrystals depend on the reaction temperature that determines the reducing ability of TEG to influence the reaction kinetics of reducing the precursor species and the following nucleation and growth kinetics.

The elemental compositions of the composite samples synthesized at different temperatures are plotted in Figure 4b and Table S3. The atomic concentrations of C (51.7 ± 3.0%), Cu (18.3 ± 1.9%), and O (30.0 ± 2.1%) remain almost constant up to 300 °C, indicating that the compositions of the Cu_2_O/rGO composite particles synthesized at temperatures ranging from 220 °C to 300 °C are essentially the same. For the sample synthesized at 330 °C, the concentration of O drops significantly to 18.0% while the concentrations of both C and Cu increase. The concentration of C increases more than that of Cu, indicating that the loss of O mainly originated from the deeper reduction of GO at 330 °C. When the reaction temperature increases to 350 °C, the synthesized sample exhibits an even lower concentration of O. The concentration of Cu increases significantly, while the concentration of C does not show an obvious change, indicating the deep reduction of Cu(OH)_2_ to Cu occurs at 350 °C. The stepwise change in the composition of the samples synthesized at different temperatures confirms that the reducing power of TEG increases with temperature and indicates that reducing GO and Cu(OH)_2_ to products with different valences requires a stepwise reducing power.

**Figure 5:**
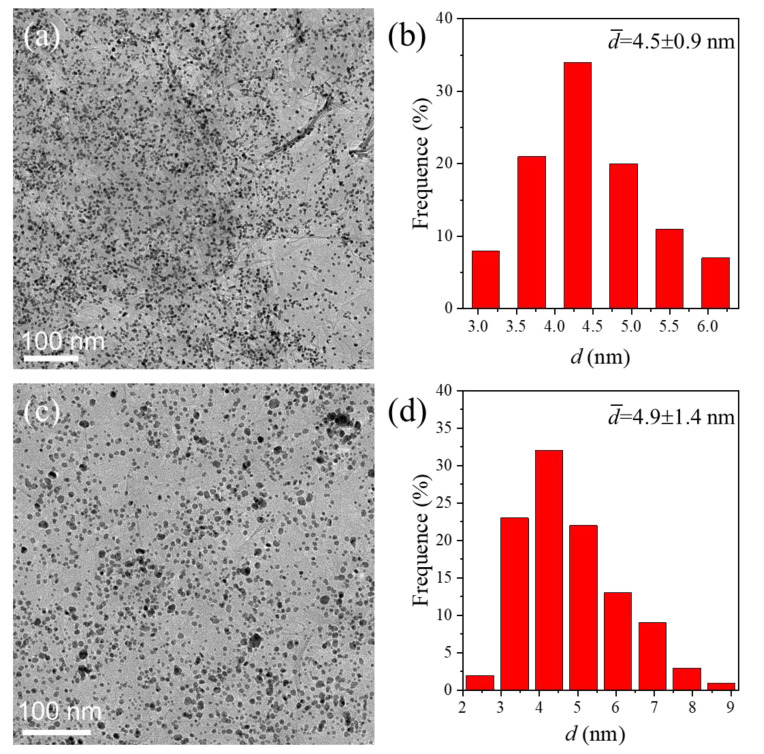
(**a**,**c**) TEM images and (**b**,**d**) statistical histograms of size distributions of the synthesized metal nanocrystals on rGO nanosheets: (**a**,**b**) Pt/rGO and (**c**,**d**) Pd/rGO. The reaction temperature was 220 °C. The statistical histograms of size distributions of the major nanocrystals were determined by analyzing the diameter (*d*) of 200 randomly selected nanoparticles.

When the GO nanosheets are soaked with solutions containing H_2_PtCl_6_ or PdCl_2_, platinum nanocrystals dispersed on rGO nanosheets (Pt/rGO) or palladium nanocrystals on rGO nanosheets (Pd/rGO) are successfully synthesized using this method highlighted in Figure 1. TEG can reduce the metal precursors at elevated temperatures to form metal nanocrystals. The synthesis of Cu_2_O/rGO, Pt/rGO, and Pd/rGO demonstrates the feasibility and generality of continuous flow reactors in synthesizing rGO-supported nanocrystals. Figure 5 presents TEM images and size distribution histograms of the synthesized metal/rGO composites, Pt/rGO (Figure 5a,b; Figure S3) and Pd/rGO (Figure 5c,d; Figure S4). Regardless of the composition of the nanoparticles, they uniformly disperse on the rGO nanosheets, and exhibit sizes smaller than 5 nm. The ultrafine size and high dispersity of the metal nanocrystals imply that the metal/rGO composites synthesized using the microfluidic reactor with liquid–solid segmented flow can expose large surface areas of metals to benefit applications such as catalysis. The synthesis solutions lack surfactant stabilizers, ensuring the clean surfaces of the metal nanocrystals to benefit catalysis. The uniform distribution of small metal nanocrystals on the rGO nanosheets indicates that the interactions between the metal nanocrystals and the rGO nanosheets are strong enough to prevent the detachment of nanocrystals from the rGO nanosheets. The stability of the metal nanocrystals can be significantly improved on the rGO nanosheets.

## 4. Conclusions

Spontaneous phase segregation of agglomerated rGO solid plugs in microfluidic liquid flow during nanoparticle synthesis reactions establishes a continuous liquid–solid segmented flow in a capillary tube reactor. The segmented flow minimizes the possibility of clogging the capillary tube, which originates from the unavoidable adsorption of nanoparticles on the inner wall of the capillary tube. The GO nanosheets dispersed in TEG are reduced to rGO nanosheets at elevated temperatures, changing the nanosheets from hydrophilic to hydrophobic. The hydrophobic rGO nanosheets repel TEG solvent to agglomerate into solid segments. When the GO nanosheets adsorb precursor species that can transform into nanoparticles through a reaction with TEG at elevated temperatures, the nanoparticles are wrapped in the agglomerated rGO segments to prevent possible sintering and ripening of the nanoparticles. All these unique features of the microfluidic synthesis method presented in this paper benefit the uniform dispersion of nanoparticles on the rGO nanosheets. The composition of the rGO-supported nanoparticles can be easily tuned by choosing the precursor species and the temperature, which determines the reducing power of TEG. Because of the absence of a surfactant in the synthesis solution, the synthesized rGO-supported nanoparticles expose clean and large-area surfaces to benefit applications such as catalysis. The successful synthesis of Cu_2_O nanocubes of different sizes, Pt, and Pd nanocrystals sheds light on the promise of continuous-flow microfluidic synthesis in capillary tube reactors.

## Data Availability

The data generated from this research are available from the authors.

## Supplementary Materials

The following supporting information can be downloaded at: https://www.mdpi.com/article/10.3390/nano12234315/s1, Schematic S1: Synthetic process of Cu_2_O nanocrystals on reduced graphene oxide (rGO) nanosheets; Figure S1: XRD patterns of the CuO/GO composite particles synthesized at different reaction temperatures when using water as the solvent; Figure S2: Typical TEM image of copper precursor supported on the GO nanosheets and the corresponding EDX mapping of Cu, C, and O; Figure S3: (a) Typical TEM image and (b) selected area electron diffraction (SAED) pattern of the Pt/rGO composite particles. The uniform concentric circles in the SAED pattern are indexed with the characteristic crystalline lattice of Pt; Figure S4: (a) Typical TEM image and (b) selected area electron diffraction (SAED) pattern of the Pd/rGO composite particles. The uniform concentric circles in the SAED pattern are indexed with the characteristic crystalline lattice of Pd; Table S1: Spontaneous agglomeration of rGO nanosheets as a function of temperature; Table S2: Full width at half maximum (FWHM) of Cu_2_O (111) XRD peaks; Table S3: Atomic compositions of the Cu_2_O/rGO samples.
