# Supplementary material for: Spontaneous Phase Segregation Enabling Clogging Aversion in Continuous Flow Microfluidic Synthesis of Nanocrystals Supported on Reduced Graphene Oxide
Source: Nanomaterials (Basel). 2022 Dec 5;12(23):4315. doi: 10.3390/nano12234315 (PMC9738359; doi:10.3390/nano12234315)
Supplement: Supplementary file 1 [file nanomaterials-12-04315-s001.zip › nanomaterials-2030106-supplementary.pdf]

nanomaterials

Supplementary Materials

Spontaneous Phase Segregation Enabling Clogging Aversion in Continuous Flow Microfluidic Synthesis of Nanocrystals Supported on Reduced Graphene Oxide

Dumei Wang [1], Dongtang Zhang [1,*], Yanan Wang [1], Guangsheng Guo [1,2], Xiayan Wang [1] and Yugang Sun [3,*]

[1] Center of Excellence for Environmental Safety and Biological Effects, Beijing Key Laboratory for Green Catalysis and Separation, Department of Chemistry and Biology, Beijing University of Technology, Beijing 100124, China

[2] Minzu University of China, Beijing 100081, China

[3] Department of Chemistry, Temple University, 1901 North 13th Street, Philadelphia, PA 19122, USA

* Correspondence: zhangdongtang@bjut.edu.cn (D.Z.); ygsun@temple.edu (Y.S.)

1. Synthetic Methods

1.1 Synthesis of graphene oxide (GO) nanosheets

GO nanosheets were synthesized using the Hummers' method. In a typical synthesis, 1 g of graphite flakes was grounded with 20 g of NaCl for 30 min to break into fine powder. The powder was transferred to a vacuum filtration apparatus and washed with copious amounts of water to remove NaCl. The remaining graphite powder was dried in an oven at 80 °C overnight. The dry powder was transferred to a 250-ml round-bottom flask, to which 23 ml of concentrated sulfuric acid was added carefully and slowly under magnetic stirring. The suspension was continuously stirred at room temperature for 24 h. To this suspension was added 100 mg of $NaNO_3$ salt that was dissolved in 5 min. 6 g of $KMnO_4$ powder was then added slowly. After $KMnO_4$ powder was dissolved, the suspension was continuously stirred for 90 min. The suspension contained high-concentration oxidizing components. Diluting the suspension with water required very careful operation, i.e., multiple step slow addition of small amount of water. Specifically, repeating the cycle of adding 3 mL of water and waiting for 5 min until 40 mL of water was added. Stirring the diluted suspension continued for 50 min. After 140 ml of water and 10 ml of 30% H_2O_2 were added to the suspension, repeating the cycle of centrifugation and washing with 5% HCl solution collected GO solid. The GO solid was re-dispersed in 100 mL of water and sonicated for 30 min to exfoliate GO into GO nanosheets. The suspension was subjected to centrifugation at 5000 rpm for 5 min only. The precipitate composed of unexfoliated GO was discarded, and the brown homogenous supernatant containing highly hydrophilic GO nanosheets was collected and dried to recover the GO nanosheets.

1.2 Synthesis of Pt or Pd nanoparticles on reduced graphene oxide (rGO) nanosheets

Typically, 10 mg of GO nanosheets were first dispersed in 20 mL of ethylene glycol with the assistance of ultrasonication for 30 min. After the GO nanosheets are dispersed, 0.10 mmol of corresponding metal precursor was added to this suspension, which was stirred for 1 h. $H_2PtCl_6·6H_2O$ and $PdCl_2$ were used as precursors for Pt nanocrystals and Pd nanocrystals, respectively. The synthesis procedure was similar to the procedure for synthesizing Cu_2O/rGO composites as described in the Experimental Section. The reaction temperature was 220 °C. The products were collected by a centrifugation process, and then washed with ethanol. The products (Pt/rGO and Pd/rGO) were dried at 60 °C in an oven overnight.

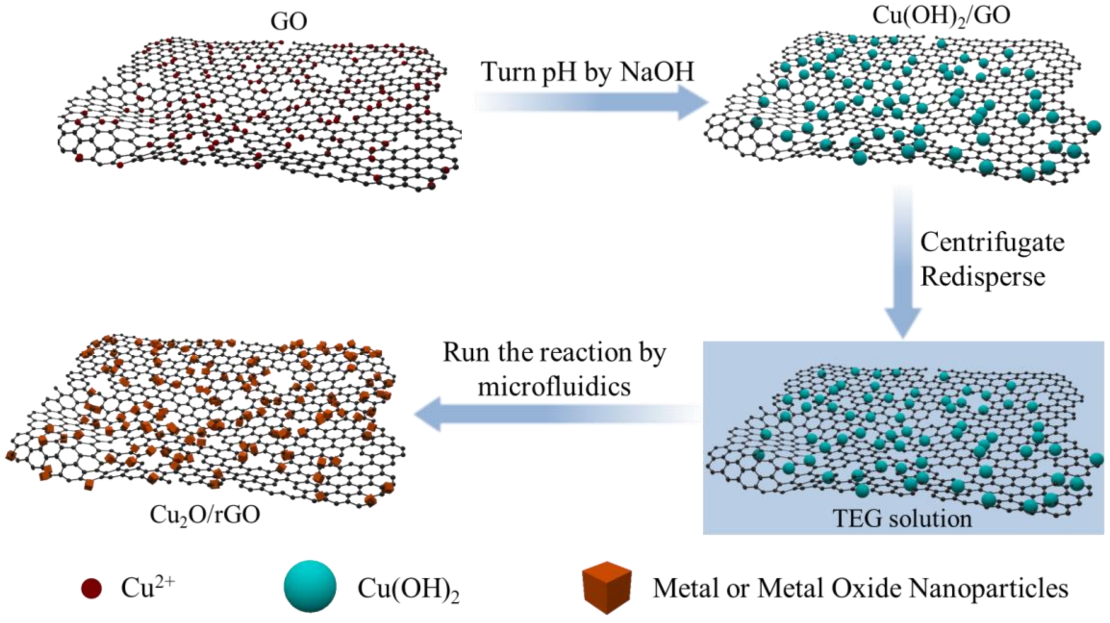

Schematic S1. Synthetic process of Cu_2O nanocrystals on reduced graphene oxide (rGO) nanosheets

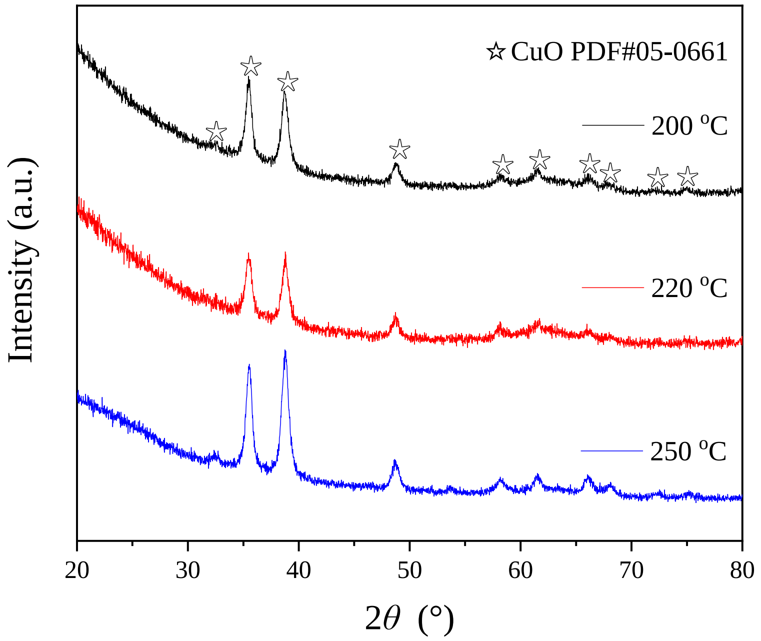

Figure S1. XRD patterns of the CuO/GO composite particles synthesized at different reaction temperatures when using water as the solvent.

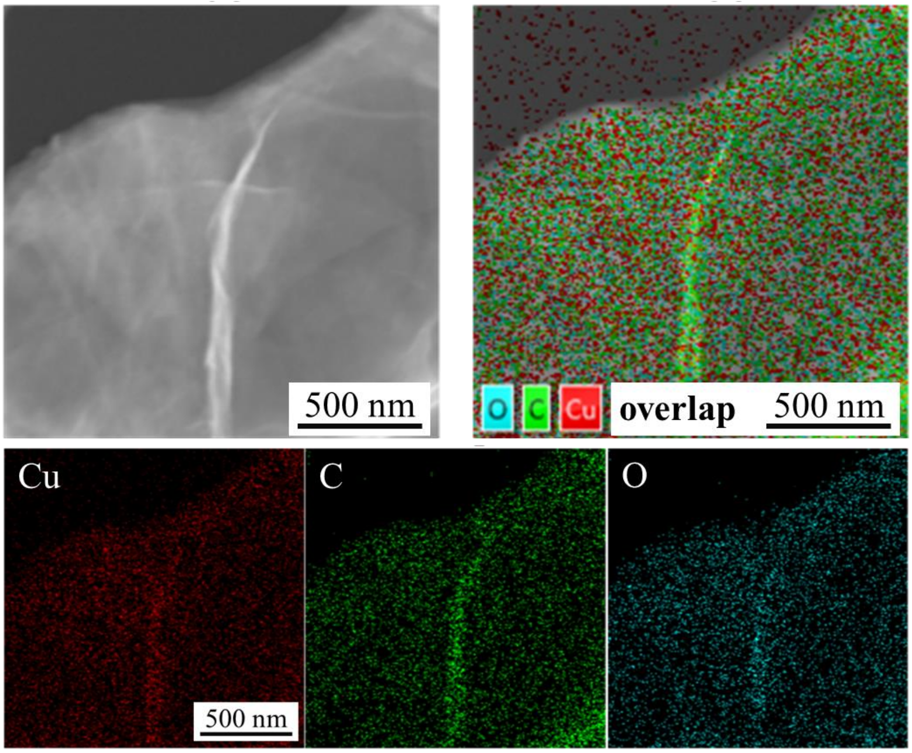

Figure S2. Typical TEM image of copper precursor supported on the GO nanosheets and the corresponding EDX mapping of Cu, C and O.

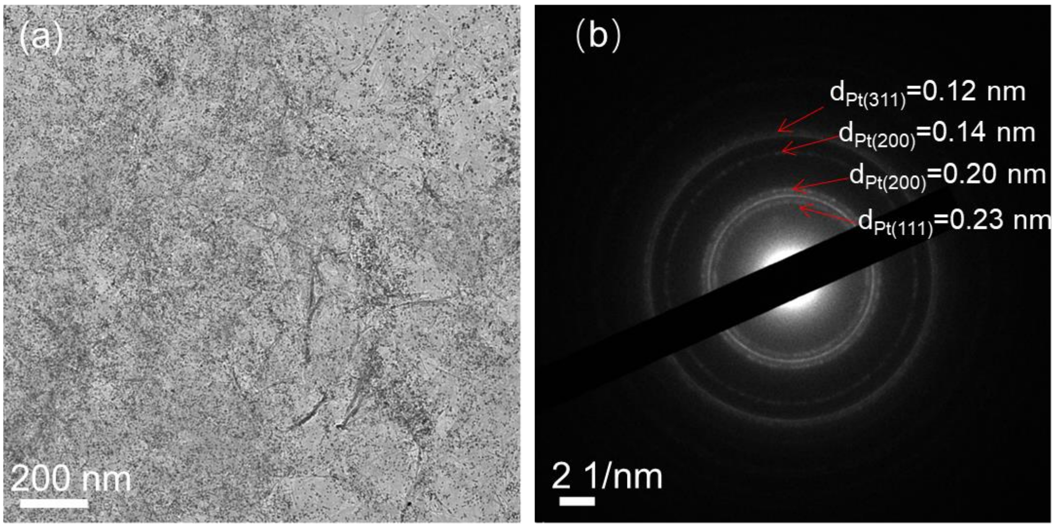

Figure S3. (a) Typical TEM image and (b) seleced area electron diffracton (SAED) pattern of the Pt/rGO composite particles. The uniform concentric circles in the SAED pattern are indexed with characteristic crystalline lattice of Pt.

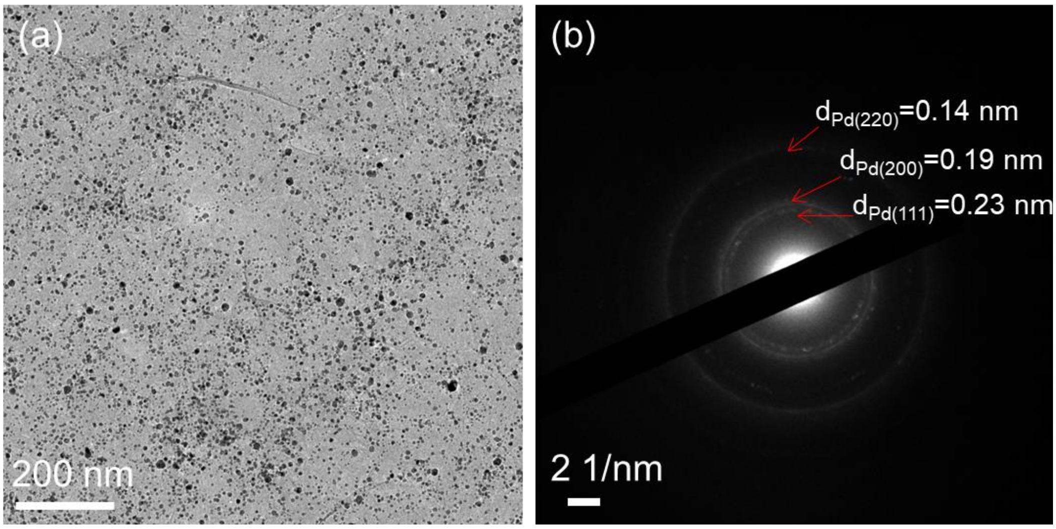

Figure S4. (a) Typical TEM image and (b) seleced area electron diffracton (SAED) pattern of the Pd/rGO composite particles. The uniform concentric circles in the SAED pattern are indexed with characteristic crystalline lattice of Pd.

Table S1. Spontaneous agglomeration of rGO nanosheets as a function of temperature

Temperature (℃)	100	150	200	250	350
Formation of agglomerated rGO solid segments	No	No	Yes	Yes	Yes

Table S2. Full width at half maximum (FWHM) of Cu_2O (111) XRD peaks

Sample	FWHM
Cu_2O/rGO-220	0.159
Cu_2O/rGO-250	0.294
Cu_2O/rGO-280	0.336
Cu_2O/rGO-300	0.340
Cu_2O/rGO-330	0.336
Cu_2O/rGO-350	0.416

Table S3. Atomic compositions of the Cu_2O/rGO samples

Sample	C (%)	Cu (%)	O (%)
Cu_2O/rGO-220	52.2	15.8	32
Cu_2O/rGO-250	51.2	18.9	29.9
Cu_2O/rGO-280	55.8	17.6	26.6
Cu_2O/rGO-300	47.4	20.9	31.6
Cu_2O/rGO-330	49	33.0	18
Cu_2O/rGO-350	43.3	43.5	13.1
